# Developing an interpretable clinical-radiomics machine learning model using whole transition zone MRI analysis for improving diagnosis of transition zone prostate cancer

**DOI:** 10.3389/fonc.2026.1716482

**Published:** 2026-02-11

**Authors:** Jinhan Yang, Ningning Jiang, Yongsheng Zhang, Liqin Yang, Lu Xia, Yue Ren, Huijing Xu, Zhiping Li, Junguang Wang, Feng Cui

**Affiliations:** 1 Department of Radiology, Hangzhou Traditional Chinese Medicine Hospital Affiliated to Zhejiang Chinese Medical University, Hangzhou, Zhejiang, China; 2 Department of Radiology, Ningbo Yinzhou No. 2. Hospital, Ningbo, Zhejiang, China

**Keywords:** benign prostatic hyperplasia, biparametric MRI, machinelearning, SHapley additive exPlanation, transition zone prostate cancer

## Abstract

**Objectives:**

Transition zone prostate cancer (TZ-PCa) presents significant diagnostic challenges due to overlapping imaging features with benign prostatic hyperplasia (BPH). This study aimed to develop and externally validate an interpretable clinical-radiomics model that integrates biparametric MRI (bp-MRI; T2-weighted imaging (T2WI) and apparent diffusion coefficient (ADC)) features with clinical variables to improve the diagnostic accuracy of TZ-PCa.

**Methods:**

A total of 280 pathologically confirmed cases from two institutions were retrospectively analyzed. Patients from Center A (n=203) were divided into a training set (n=142) and an internal validation set (n=61), while patients from Center B (n=77) constituted an external validation set. The whole transitional zone on the slice corresponding to the tumor’s largest diameter was delineated as a single-slice region-of-interest (ROI). Radiomics features were extracted and used to train six machine learning algorithms to construct single-sequence (T2WI or ADC) and combined-sequence (ADC+T2WI) models. The best radiomics model was then combined with independent clinical characteristics to construct a clinical-radiomics model. Model performance was evaluated by Receiver Operating Characteristic (ROC) analysis, and clinical utility was assessed with calibration and decision curve analyses (DCA). The interpretability of the optimal model was further examined using Shapley Additive Explanation (SHAP).

**Results:**

Multivariate logistic regression analysis identified PI-RADS score (odds ratio (OR)=3.47, 95%CI 1.90~6.35, *P<0.001*) and total prostate specific antigen (tPSA) (OR = 1.06, 95%CI 1.01~1.12, *P=0.020*) as independent clinical predictors. The support vector machine (SVM) radiomics model using combined ADC+T2WI features achieved AUCs of 0.865 (training) and 0.850 (internal validation). The clinical-radiomics model yielded AUCs of 0.963, 0.889, and 0.829 in the training, internal validation, and external validation sets, respectively. SHAP analysis identified T2-wavelet-LLH_glszm_SmallAreaLowGrayLevelEmphasis as the most crucial feature.

**Conclusion:**

The proposed clinical-radiomics model demonstrated the best diagnostic performance for differentiating TZ-PCa from BPH across bio-centers. Combining the SHAP algorithm with the model enhances interpretability and may assist clinicians in making more precise diagnostic and treatment decisions.

## 1 Introduction

Prostate cancer (PCa) is the second most common malignancy in men worldwide (1, 2). Approximately 30% of tumors originate in the transition zone (TZ), an anatomical region that is also frequently by benign prostatic hyperplasia (BPH) (3). Differentiating TZ-PCa from BPH is challenging because of overlapping clinical symptoms and elevated prostate-specific antigen (PSA) levels, and such diagnostic uncertainty can contribute to failure of active surveillance in some patients (4, 5).

Given ongoing debate regarding the added value of dynamic contrast-enhanced MRI for TZ-PCa, the Prostate Imaging Reporting and Data System version 2.1 (PI-RADS v2.1) guideline introduces biparametric MRI (bp-MRI), typically comprising T2-weighted imaging (T2WI) and diffusion-weighted imaging (DWI) with derived apparent diffusion coefficient (ADC) maps, to streamline prostate MRI protocols. For TZ assessment, T2WI is the dominant sequence (6). However, TZ-PCa often demonstrates homogeneous low T2 signal, with indistinct margins and lenticular morphology that overlap with BPH, making differentiation solely through visual inspection challenging (7). Additionally, the subjectivity of PI-RADS v2.1 scoring further limits inter-observer agreement (8).

Radiomics enables quantitative characterization of image phenotypes beyond visual assessment by extracting high-dimensional features (9–11). Thus, histopathologic differences between TZ-PCa and BPH can be captured by radiomics features. Most prior research has primarily focused on analyzing lesion-specific regions of interest (ROIs), which require accurate lesion localization and are susceptible to boundary ambiguity in small or occult TZ lesions (12). To improve feasibility and reproducibility, we adopted a simplified method in which the entire TZ on the axial slice corresponding to the tumor’s largest diameter was delineated as the ROI on both T2WI and ADC. This approach avoided the need for pre-identification of lesions and reducing contouring uncertainty.

A major barrier to clinical adoption of machine learning models is limited interpretability. The Shapley Additive Explanations (SHAP) method provides global and local attributions of feature contributions, clarifying how predictors influence individual and cohort-level outputs and thereby increasing clinical trust in model decisions (13–15). Integrating SHAP with radiomics may thus enhance transparency without sacrificing performance.

Accordingly, we aimed to develop and validate an interpretable clinical-radiomics model that integrates bp-MRI (T2WI and ADC) features with clinical predictors to differentiate TZ-PCa from BPH, and to employ SHAP to intuitively interpret the predicted process for clinical application.

## 2 Materials and methods

### 2.1 Patient population

**Figure 1:**
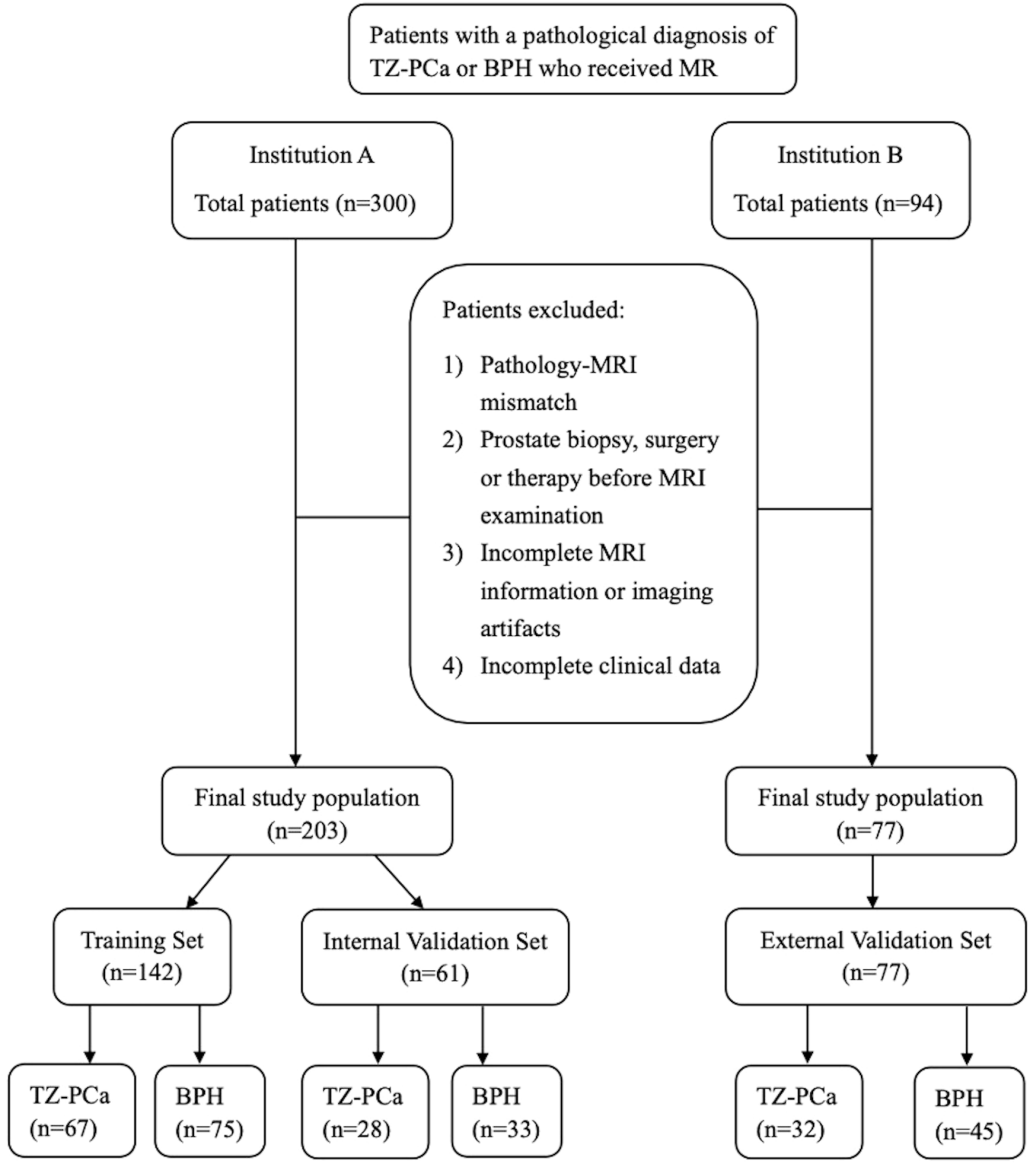
Flowchart of patient selection.

This retrospective, two center study was approved by the institutional review boards of both institutions, and the requirement for informed consent was waived. Consecutive patients with pathologically confirmed TZ-PCa or BPH were screened at Center A between July 2023 and June 2024, and at Center B between November 2016 and November 2024. Of 300 candidates at Center A, 203 met the inclusion criteria: (1) no prior prostate biopsy, surgery, radiotherapy, chemotherapy, endocrine therapy, or cryotherapy before bp-MRI, and pathological results must be collected in less than 2 months following imaging; (2) TZ-PCa confirmed by surgical pathology with more than 70% of tumor volume located in the transition zone and volume >0.5 cm³; (3) BPH confirmed by surgical pathology with low or iso-low signal nodules within the transition zone on T2WI (12). Exclusion criteria were incomplete MRI data or clinical data and nondiagnostic image quality. Patients from Center A were randomly assigned (stratified 7:3) to a training set (n=142) and an internal validation set (n=61). An independent external validation set (n=77) was from Center B. A flowchart illustrating patient recruitment is presented in **Figure 1**.

### 2.2 Clinical data

Clinical data were retrieved from the electronic medical records, including age, PI-RADS score, total PSA (tPSA), free PSA (fPSA), and peripheral blood counts (neutrophils, platelets, lymphocytes, monocytes). PI-RADS scoring followed version 2.1 and was independently assigned by two radiologists (7 and 14 years of experience in prostate MRI diagnosis) who were blinded to pathology. In case of disagreement, the final score was determined by the radiologist with 14 years of experience.

### 2.3 MRI image acquisition

At Center A, bp-MRI was performed on a 1.5T GE SIGNA Voyager system, while at Center B, on a 3.0T GE Discovery 750. The field of view covered the entire prostate and seminal vesicle. The bp-MRI protocol included axial and sagittal T2WI, and axial DWI sequences. ADC maps were automatically generated by each MRI system using a standard mono-exponential model on the scanner. The full image protocols and detailed parameters are provided in Supplementary Materials (**Supplementary Table S1**).

### 2.4 Image preprocessing and segmentation

**Figure 2:**
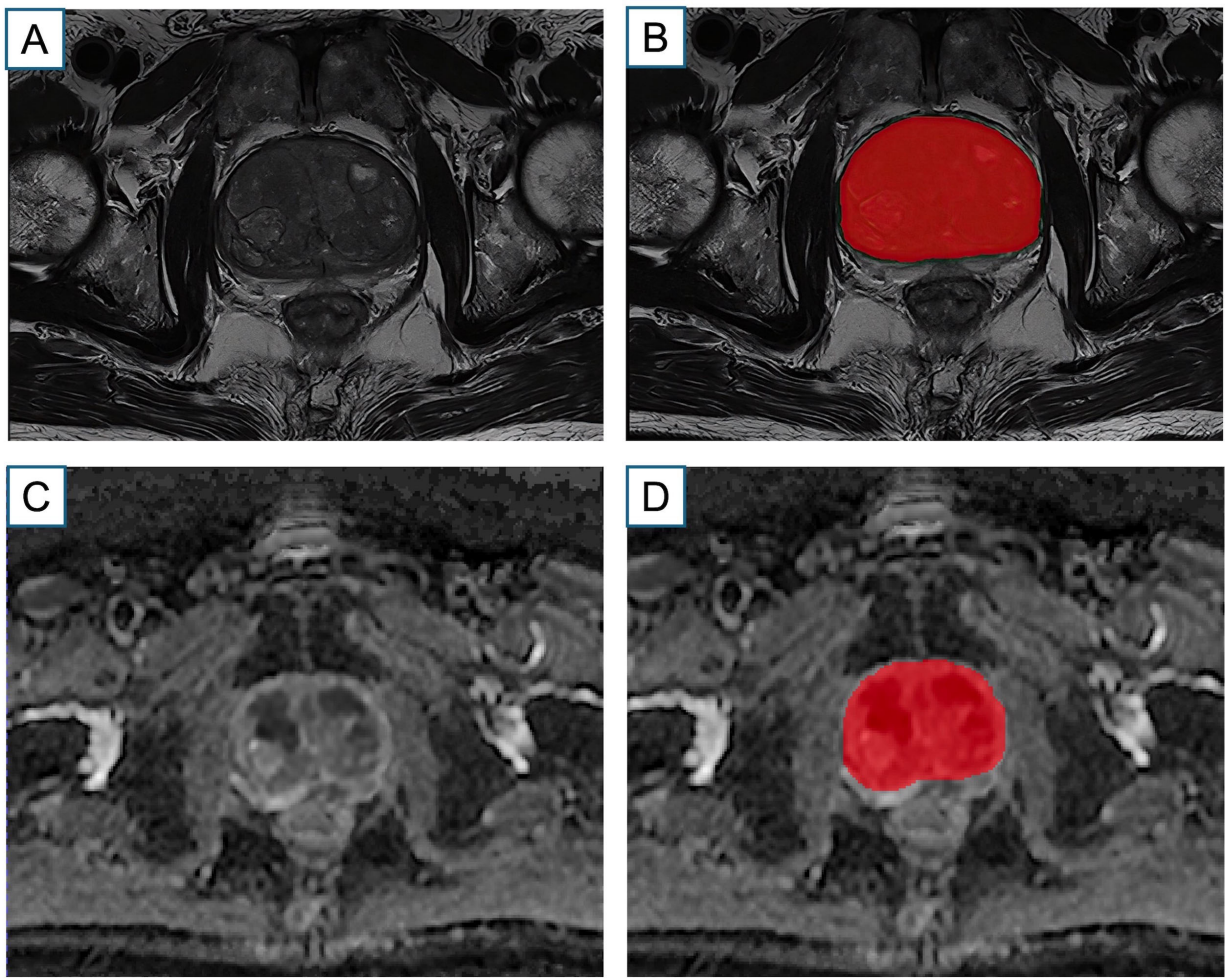
Schematic Diagram of ROI Delineation. Example axial T2-weighted imaging (T2WI) **(A)**, apparent diffusion coefficient (ADC) map **(C)** from a patient with TZ-PCa. Single-slice whole TZ-ROIs are outlined in the T2WI and ADC **(B, D)**.

Following PI-RADS v2.1 guidelines and prior evidence highlighting ADC differences between TZ-PCa and BPH, we analyzed axial T2WI and ADC images for ROI segmentation and radiomics feature extraction. To reduce inter-site variability, N4 bias field was applied, followed by gray-level intensity normalization, enhancing comparability across datasets. Image preprocessing was performed by resampling the images with a resolution of 1 × 1 × 1 mm^3^ through the linear interpolation method and by discretizing and normalizing the image gray level to order 25. For each case, the whole TZ on the axial slice corresponding to the tumor’s largest diameter was manually delineated as the ROI on both T2WI and ADC using ITK-SNAP open-source software (Version 3.80, http://www.itk-snap.org). Initial contours were drawn by a radiologist with two years of experience and reviewed by a senior genitourinary radiologist with 14 years of experience. All segmentations were performed manually to ensure consistency. The segmentation process is illustrated in **Figure 2**.

### 2.5 Radiomics feature extraction and selection

Radiomics features were extracted using Python 3.7.0 (https://www.python.org/) and the Pyradiomics package. The extracted features included shape, first-order intensity, and texture features (gray-level co-occurrence matrix (GLCM), gray-level run-length matrix (GLRLM), gray-level size-zone matrix (GLSZM), and gray-level dependence matrix (GLDM)), together with features derived through wavelet transformations. To assess reproducibility, features with intraclass correlation coefficient (ICC) ≥ 0.75 were selected. Features with a normal distribution were analyzed using independent sample t-tests, whereas non-normally distributed features were assessed with the Mann-Whitney *U* test. A two-tailed *P* value < 0.05 was considered statistically significant. To address potential multicollinearity and minimize feature redundancy, correlation analysis was subsequently performed. Finally, all features were Z-score standardized, and the least absolute shrinkage and selection operator (LASSO) with 10-fold cross-validation was applied to select the optimal regularization parameter (λ) for model training (**Supplementary Figures S1, S2**).

### 2.6 Model construction and evaluation

Univariate analysis was first performed to identify clinical features with statistically significant differences between the TZ-PCa and BPH groups across the three datasets. Clinical features that exhibited significant differences were then incorporated into both univariate and multivariate logistic regression models to identify independent risk factors for TZ-PCa. Based on the results, a clinical model was developed using these independent clinical predictors.

Using the reduced feature subsets from T2WI and ADC, we trained six machine learning algorithms, including support vector machine (SVM), random forest (RF), K-nearest neighbors (KNN), stochastic gradient descent (SGD), extreme gradient boosting (XGBoost), and light gradient boosting machine (LightGBM) to construct sequence-specific (T2WI or ADC) and combined (ADC+T2WI) radiomics models. The Center A dataset was split into a training set and an internal validation set. The internal validation set was used for model selection (i.e., selecting the optimal sequence and classifier based on AUC), whereas the external validation cohort from Center B was reserved for independent general is ability assessment.

Training-set AUCs for the models were calculated using out-of-fold (OOF) predictions from 5-fold cross-validation. This approach provides a less biased estimate of model performance on unseen data.

**Figure 3:**
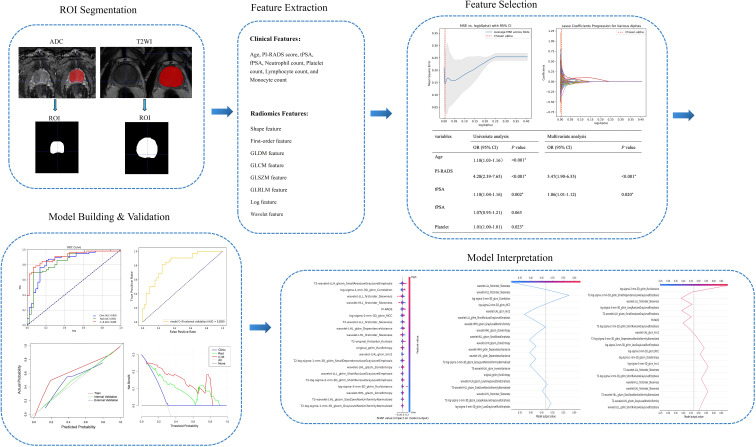
Workflow of this research.

Following the selection of the optimal radiomics model, independent clinical predictors (PI-RADS score and tPSA) were integrated with the radiomics signature to develop a combined clinical–radiomics model using SVM. Performance was evaluated using ROC analysis with AUC and 95% confidence intervals in the training, internal validation, and external validation sets. Calibration was assessed by calibration plots and the Brier score, and clinical utility by decision curve analysis (DCA). Confusion matrices and threshold-specific metrics were reported. Model interpretability was examined using SHAP to provide global (bees-warm) and local (decision plot) attributions. Finally, to verify the combined model’s generalizability, external validation was used to validate the diagnostic performance. The entire workflow of this study is shown in **Figure 3**.

### 2.7 Statistical analysis

All statistical analyses were conducted in SPSS (v23.0) and Python (scikit-learn, XGBoost, LightGBM). Normality was tested using the Kolmogorov-Smirnov test. Continuous variables were summarized as mean ± standard deviation (SD) and compared using the t-test or Mann-Whitney *U* test, as appropriate. Categorical variables were compared using the chi-square test or Fisher’s exact test.Two-sided *P* < 0.05 indicated statistical significance.

## 3 Results

### 3.1 Patient characteristics

A total of 280 patients were analyzed: 142 in the training set (TZ-PCa, n=67; BPH, n=75), 61 in the internal validation set (TZ-PCa, n=28; BPH, n=33) and 77 in the external validation set (TZ-PCa, n=32; BPH, n=45). Patient characteristics are summarized in **Table 1**. Statistically significant differences in PI-RADS scores and tPSA levels were observed between the TZ-PCa and BPH groups across all cohorts *(P* < 0.05). Age showed significant differences in both the training and external validation sets, while fPSA and platelet count differed significantly only in the training set *(P* < 0.05).

**Table 1:** Clinical characteristics of TZ-PCa and BPH patients.

Characteristics	Training set (142)		*P* value	Internal validation set (61)		*P* value	External validation set (77)		*P* value
	BPH (N=75)	TZ-PCa (N=67)		BPH (N=33)	TZ-PCa (N=28)		BPH (N=45)	TZ-PCa (N=32)	
PI-RADS			<0.001*			<0.001*			<0.001*
1	1 (1.3%)	0 (0%)							
2	22 (29.3%)	5 (7.5%)		11 (33.3%)	1 (3.6%)		1 (2.2%)	0 (0%)	
3	45 (60%)	28 (41.8%)		18 (54.5%)	13 (46.4%)		26 (57.8%)	3 (9.4%)	
4	6 (8%)	25 (37.3%)		4 (12.1%)	8 (28.6%)		16 (35.6%)	8 (25%)	
5	1 (1.3%)	9 (13.4%)		0 (0%)	6 (21.4%)		2 (4.4%)	21 (65.6%)	
Age, Mean ± SD	66.64±6.02	69.96±6.12	0.001*	67.64±6.79	71.14±8.29	0.074	70.18±9.36	74.53±8.03	0.036*
tPSA (ng/mL), Median (IQR)	6.38 (5.01, 8.29)	11.23 (7.63, 17.03)	<0.001*	6.78 (5.06, 8.09)	11.16 (6.96, 17.06)	0.001*	9.62 (5.87, 14.00)	19.96 (10.12, 35.59)	<0.001*
fPSA (ng/mL), Median (IQR)	0.85 (0.61, 1.29)	1.31 (0.94, 2.12)	<0.001*	0.82 (0.68, 1.03)	1.27 (0.66, 2.29)	0.071	1.73 (1.32, 3.41)	2.05 (1.49, 5.43)	0.211
Neutrophil (10^9/L), Median (IQR)	3.47 (2.71, 4.26)	3.55 (3.05, 4.12)	0.547	2.99 (2.56, 4.18)	3.49 (2.97, 4.34)	0.239	3.72 (2.94, 4.82)	3.95 (3.58, 4.92)	0.260
Platelet(10^9/L), Median (IQR)	192.00 (166.00, 224.50)	211.00 (178.50, 256.00)	0.028*	179.00 (160.00, 206.00)	191.50 (164.50,204.50)	0.894	201.00 (162.00, 225.00)	192.50 (174.00, 229.00)	0.861
Monocytes (10^9/L), Median (IQR)	0.42 (0.32, 0.57)	0.44 (0.38, 0.50)	0.830	0.36 (0.30, 0.42)	0.41 (0.35, 0.49)	0.069	0.50 (0.33, 0.61)	0.46 (0.37, 0.64)	0.729
Lymphocyte (10^9/L), Median (IQR)	1.37 (1.10, 1.81)	1.38 (1.06, 1.75)	0.775	1.18 (0.94, 1.47)	1.29 (1.05, 1.66)	0.308	1.56 (1.19, 1.82)	1.29 (0.97, 1.77)	0.144

PI-RADS, Prostate Imaging Reporting and Data System; tPSA, total prostate-specific antigen; fPSA, free prostate-specific antigen; IQR, interquartile range; SD, standard deviation. * *P* < 0.05.

### 3.2 Clinical model

In univariate analysis, age, PI-RADS score, tPSA, and platelet count were associated with TZ-PCa. In multivariate logistic regression analysis, PI-RADS score (OR = 3.47,95%CI, 1.90~6.35, *P<0.001*) and tPSA (OR = 1.06, 95%CI, 1.01~1.12, *P=0.020*) were independent risk factors for TZ-PCa (**Table 2**). A clinical model built on these variables yielded AUCs of 0.786 (95% CI, 0.704-0.857) in the training set and 0.850 (95% CI, 0.738-0.940) in the internal validation set.

**Table 2:** Results of univariate and multivariate logistic regression analysis.

Variables	Univariate analysis		Multivariate analysis	
	OR (95% Cl)	*P* value	OR (95% Cl)	*P* value
Age	1.10 (1.03-1.16)	<0.001^*^		
PI-RADS	4.28 (2.39-7.65)	<0.001^*^	3.47 (1.90-6.35)	<0.001^*^
tPSA	1.10 (1.04-1.16)	0.002^*^	1.06 (1.01-1.12)	0.020^*^
fPSA	1.07 (0.95-1.21)	0.063		
Platelet	1.01 (1.00-1.01)	0.023^*^		

OR, odds ratio; CI, confidence interval; tPSA, total prostate-specific antigen; fPSA, free prostate-specific antigen. * *P* < 0.05.

### 3.3 Radiomics model

From a total of 1130 extracted radiomics features, 11 ADC features, 16 T2WI features, and 65 combined ADC+T2WI features were ultimately retained after dimensionality reduction and selection in the training set. These retained features included 1 shape descriptor,14 first-order statistics, 14 GLDM features, 18 GLRLM features, 37 GLSZM features, and 8 GLCM features. Performance comparison of six machine learning classifiers demonstrated that the SVM model trained on combined ADC+T2WI features achieved the highest performance in differentiating TZ-PCa from BPH, with AUCs of 0.865 in the training set and 0.850 in the internal validation set. In the same settings, the specificity, sensitivity, and accuracy reached 0.800, 0.860, and 0.830 in the training sets, and 0.770, 0.840, and 0.800 in the internal validation sets, respectively (**Table 3**). On the basis of these results, this combined ADC+T2WI SVM was selected as the optimal radiomics model and further applied for constructing the combined model. The evaluation performance of the SVM Radiomics model is detailed in the Supplementary materials (**Supplementary Figure S3**).

**Table 3:** Performance of different radiomics machine learning models in differentiating TZ-PCa from BPH.

	Training set				Internal validation set			
	AUC (95%Cl)	Specificity	Sensitivity	Accuracy	AUC (95%Cl)	Specificity	Sensitivity	Accuracy
SVM model								
ADC	0.911 (0.850-0.972)	0.830	0.800	0.820	0.824 (0.731-0.916)	0.870	0.790	0.820
T2WI	0.870 (0.820-0.918)	0.830	0.750	0.780	0.764 (0.680-0.847)	0.740	0.710	0.720
ADC+T2WI	0.865 (0.801-0.930)	0.800	0.860	0.830	0.850 (0.755-0.944)	0.770	0.840	0.800
KNN model								
ADC	0.886 (0.826-0.947)	0.870	0.780	0.810	0.755 (0.664-0.847)	0.760	0.750	0.750
T2WI	0.862 (0.811-0.913)	0.790	0.780	0.780	0.742 (0.665-0.820)	0.710	0.700	0.700
ADC+T2WI	0.859 (0.807-0.918)	0.880	0.760	0.800	0.834 (0.754-0.915)	0.800	0.710	0.740
Light GBN model								
ADC	0.851 (0.794-0.909)	0.810	0.800	0.800	0.804 (0.715-0.893)	0.710	0.800	0.750
T2WI	0.792 (0.718-0.865)	0.700	0.710	0.700	0.811 (0.694-0.928)	0.690	0.750	0.720
ADC+T2WI	0.926 (0.849-1.000)	0.890	0.870	0.880	0.828 (0.712-0.944)	0.730	0.740	0.740
RF model								
ADC	0.897 (0.850-0.943)	0.830	0.860	0.850	0.816 (0.742-0.890)	0.790	0.840	0.820
T2WI	0.866 (0.824-0.907)	0.770	0.780	0.770	0.839 (0.775-0.902)	0.810	0.800	0.800
ADC+T2WI	0.908 (0.864-0.952)	0.870	0.840	0.850	0.848 (0.783-0.913)	0.740	0.730	0.740
SGD model								
ADC	0.895 (0.871-0.919)	0.830	0.800	0.820	0.817 (0.781-0.853)	0.830	0.760	0.790
T2WI	0.891 (0.865-0.916)	0.880	0.770	0.810	0.763 (0.722-0.803)	0.710	0.680	0.690
ADC+T2WI	0.928 (0.874-0.983)	0.890	0.870	0.880	0.847 (0.765-0.928)	0.700	0.820	0.770
XG Boost model								
ADC	0.836 (0.764-0.907)	0.780	0.780	0.780	0.842 (0.729-0.955)	0.790	0.840	0.820
T2WI	0.820 (0.755-0.886)	0.760	0.720	0.740	0.818 (0.714-0.922)	0.760	0.810	0.790
ADC+T2WI	0.934 (0.868-0.998)	0.910	0.850	0.870	0.810 (0.709-0.910)	0.700	0.740	0.720

### 3.4 Clinical-Radiomics combined model

**Figure 4:**
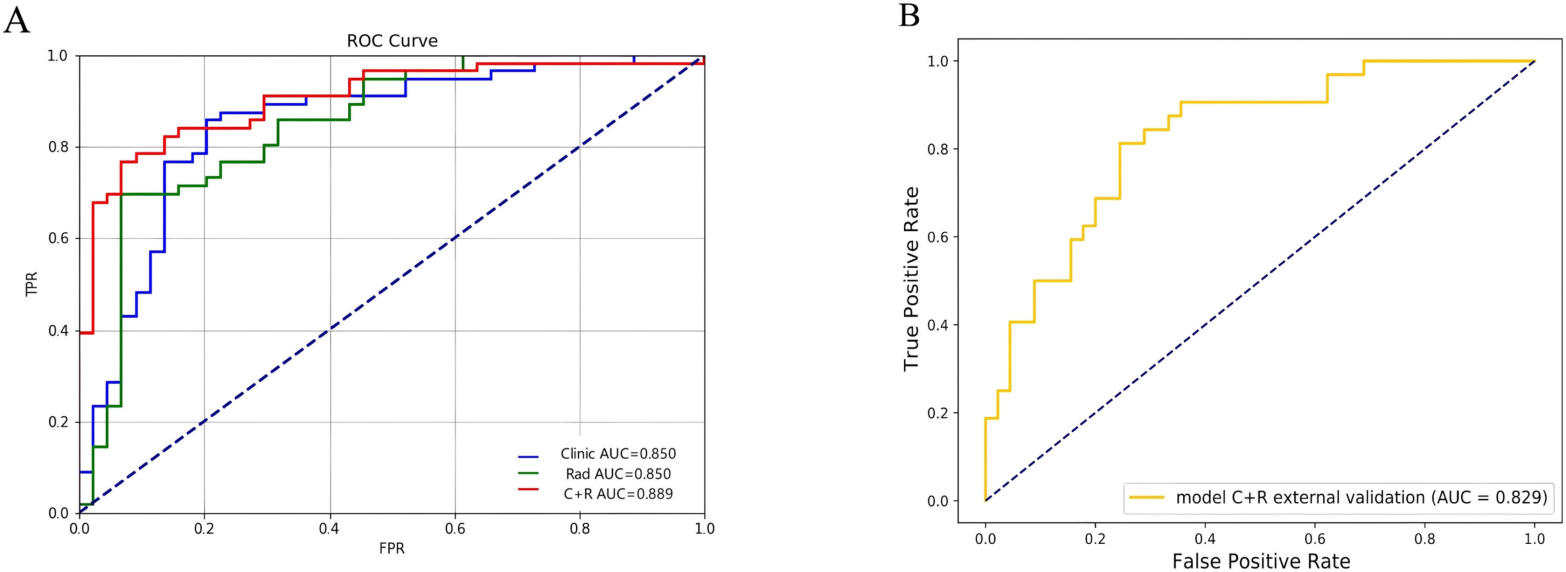
Receiver Operating Characteristic (ROC) curves for the Clinical Model, SVM Radiomics Model, and Clinical-Radiomics Combined Model in the internal validation set for distinguishing between TZ-PCa and BPH **(A)**. ROC curve for the Clinical-Radiomics Combined Model in the external validation set for distinguishing between TZ-PCa and BPH **(B)**.

**Figure 5:**
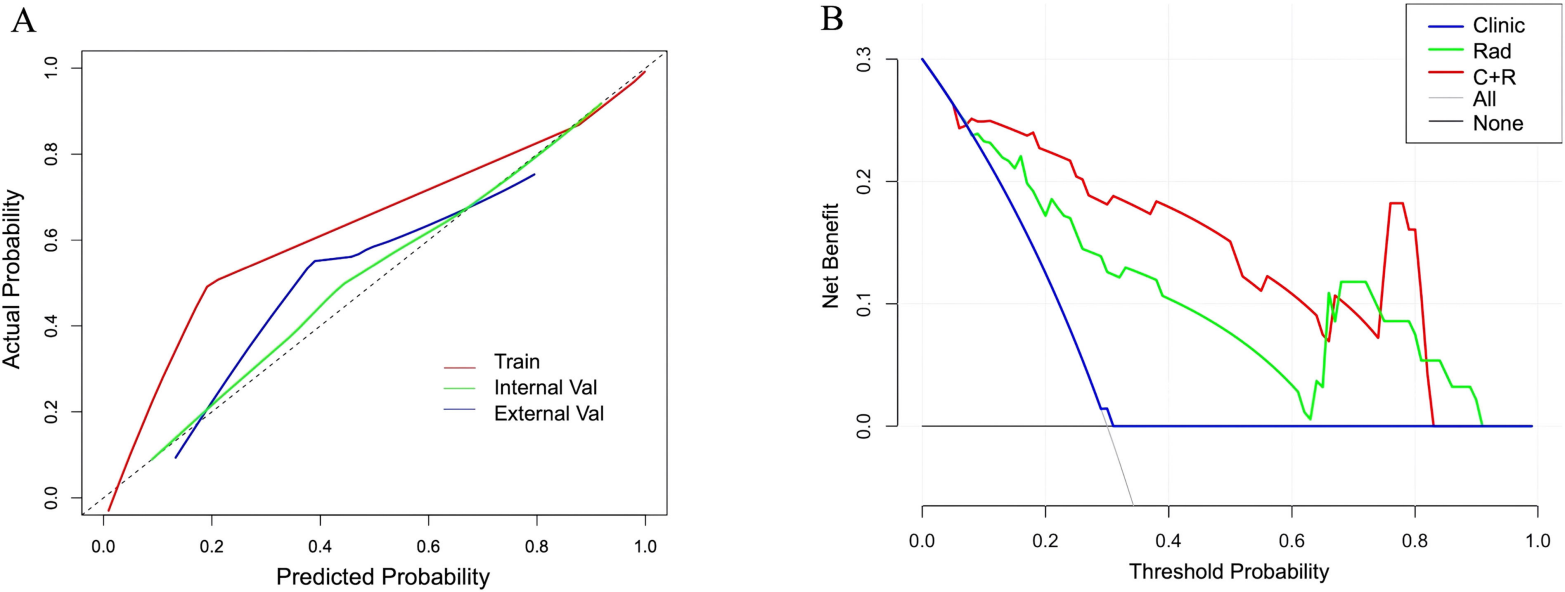
Model evaluation. Calibration curve of Clinical-Radiomics Combined Model in the training set, internal validation set, and external validation set **(A)**; Decision curves of three models in the internal validation set **(B)**.

Integrating PI-RADS score and tPSA with the combined-sequence radiomics features using an SVM classifier yielded the clinical-radiomics combined model. This model achieved an AUC of 0.963 (95% CI, 0.897-1.000) in the training set and 0.889 (95% CI, 0.786-0.991) in the internal validation set, outperforming the clinical-only and radiomics-only models (**Table 4**). In the external validation set, the clinical-radiomics model reached an AUC of 0.829, indicating good generalizability. ROC comparisons are shown in **Figures 4A**, **B**. Calibration curves exhibited good agreement between predicted and observed actual TZ-PCa outcomes across datasets (**Figure 5A**). DCA plots showed that, to a large extent, the net clinical benefit of the Combined model was higher than that of the Clinical model and the Radiomics model (**Figure 5B**).

**Table 4:** Performance of the clinical model, SVM radiomics model, and clinical-radiomics combined model in the training set and internal validation set.

	Model	AUC (95%Cl)	Specificity	Sensitivity	Accuracy
training set	Clinical model	0.786(0.704-0.857)	0.710	0.800	0.750
	Radiomics model	0.865(0.801-0.930)	0.800	0.860	0.830
	Combined model	0.963(0.897-1.000)	0.980	0.900	0.940
internal validation set	Clinical model	0.850(0.738-0.940)	0.820	0.790	0.800
	Radiomics model	0.850(0.755-0.944)	0.770	0.840	0.800
	Combined model	0.889(0.786-0.991)	0.810	0.820	0.820

AUC, area under the curve; CI, confidence interval.

### 3.5 SVM combined model for SHAP

**Figure 6:**
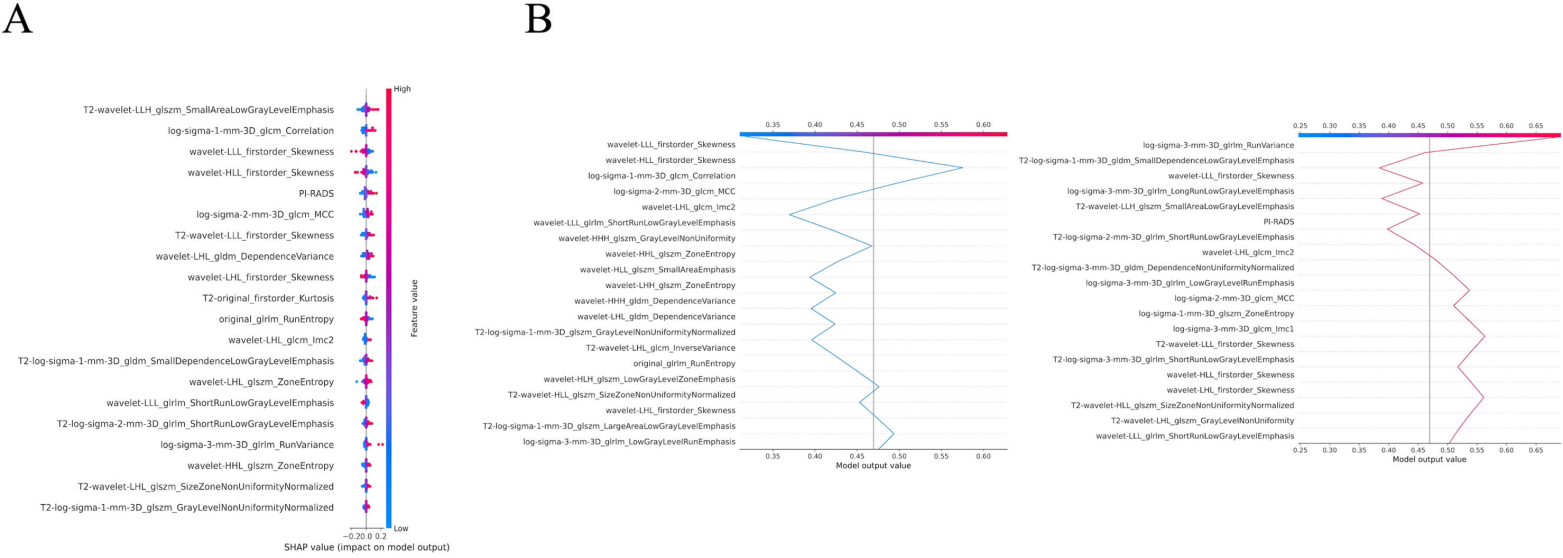
Visualization of the model through SHAP. The SHAP bees-warm plot shows the weight of the twenty most important characteristics in the model and the positive or negative effects of each feature on the prediction probability through red and blue colors **(A)**; The SHAP decision plot shows the impact process of each significant feature on the final diagnostic probability **(B)**.

The optimal model was interpreted using the SHAP algorithm to facilitate model interpretation and potential clinical application. The overall and individual Shapley values were calculated for the SVM combined model. In the global analysis, the SHAP bees-warm plot (**Figure 6A**) shows the twenty most significant features, with red and blue colors indicating positive or negative contributions to the diagnostic probability, respectively. Among these, T2-wavelet-LLH_glszm_SmallAreaLowGrayLevelEmphasis exhibited the highest weight, exerting a positive effect on the probability of diagnosing TZ-PCa in the combined model. In the individual analysis, the SHAP decision plot (**Figure 6B**) shows how each significant feature sequentially contributed to the final diagnostic probability.

## 4 Discussion

In this two-center study, we developed an interpretable clinical-radiomics model that integrates bp-MRI (T2WI and ADC) features with clinical parameters to differentiate TZ-PCa from BPH. The combined model outperformed the clinical-only and radiomics-only models in the internal validation set (AUC, 0.889) and maintained good discrimination in the external set (AUC, 0.829). Calibration and decision-curve analyses further supported its potential clinical utility, while SHAP provided case-level and cohort-level explanations of feature contributions.

The diagnostic accuracy of conventional imaging based on the PI-RADS scoring system remains limited by inter-observer variability and the inherent subjectivity of image interpretation (16). Radiomics provides quantitative features of tumor heterogeneity and underlying biological characteristics that are not readily appreciable on conventional imaging, thereby enhancing diagnostic efficiency (17). Higher heterogeneity in TZ-PCa allows radiomics features to detect the differences overlooked by visual assessment. This heterogeneity contributes to the good performance of our model in differentiating TZ-PCa from BPH. Recent multicenter studies have also shown the value of bp-MRI radiomics for TZ-PCa, including better diagnosis of equivocal PI-RADS 3 lesions and improved risk stratification when combined with clinical variables (18, 19). Previous studies have shown that radiomics models based on bp-MRI can effectively diagnose PCa. For instance, Chen et al. developed radiomics models using ADC, T2WI, and combined ADC+T2WI sequences to diagnose clinically significant PCa, achieving AUC values of 0.985, 0.982, and 0.999, respectively, and highlighted the superior performance of the combined sequence model over single-sequence models, which is consistent with the conclusion of our study (20). Wu et al. reported that logistic regression and SVM models incorporating ADC percentile features as well as T2WI-based shape and texture features achieved high diagnostic accuracy for TZ-PCa (21). As a machine learning algorithm, SVM is particularly advantageous for small-sample, high-dimensional datasets, offering stability, reliability, and robust generalization (22). Although the sample size in our study was only slightly larger than that in prior investigations, the SVM-based model nonetheless demonstrated superior performance, underscoring its suitability for improving the stability and generalization ability of radiomics models in this clinical context. Building on these imaging-derived insights, combination with clinical biomarkers further improves diagnostic performance.

In addition to radiomics, PSA, including both tPSA and fPSA, is widely utilized as a biomarker for the detection and monitoring of prostate cancer. However, elevated PSA levels can also be observed in patients with PCa or BPH, thereby limiting its specificity. To address this limitation, the PI-RADS scoring system was introduced, and multiple studies have confirmed the predictive value of PI-RADS v2 (23, 24). Nevertheless, a meta-analysis reported substantial variability in its positive predictive values (PPVs), ranging from 0.31 to 0.95 (25). More recently, Lee et al. demonstrated that PI-RADS v2.1 provides diagnostic performance comparable to PI-RADS v2, but at the expense of lower specificity, potentially leading to more unnecessary biopsies (26). These findings indicate PI-RADS scores alone are insufficient to reliably identify patients who can safely avoid biopsies. In our study, univariate and multivariate logistic regression analyses identified the PI-RADS score and tPSA as independent risk factors for TZ-PCa. Building on this, we developed a combined clinical-radiomics model by integrating radiomics features with these clinical predictors. In the internal validation set, the combined model (AUC = 0.889) outperformed both the clinical model (AUC = 0.850) and the radiomics-only model (AUC = 0.850) in differentiating TZ-PCa from BPH. By integrating imaging-derived quantitative features with clinical features, the combined model leverages complementary sources of information, thus enhancing diagnostic accuracy. This integrative approach holds significant clinical promise, as radiomics-assisted models may serve as effective tools for clinical decision-making, optimizing diagnostic pathways and reducing unnecessary prostate biopsies.

In addition, most previous imaging studies on TZ-PCa have been limited to single-center cohorts (27). In contrast, our study incorporated external validation, where the combined model achieved an AUC of 0.829, thereby confirming its robust generalizability. The observed reduction in AUC in external validation from 0.889 to 0.829 (6.0%) may reflect inter-center variability in MRI acquisition protocols. This finding highlights the importance of standardizing bp-MRI acquisition parameters in future multicenter studies, thereby improving reproducibility and clinical applicability.

Beyond predictive performance, model interpretability is essential for clinical translation. Interpretable machine learning techniques, such as SHAP, have been increasingly applied to mitigate the “black box” phenomenon (28). By quantifying the marginal contribution of each feature and visualizing their overall and individual impacts, SHAP improves transparency and can increase clinicians’ confidence in adopting such models. In our analysis, SHAP revealed that the top twenty features contributed substantially to diagnostic decision-making, with the T2-wavelet-LLH_glszm_SmallAreaLowGrayLevelEmphasis emerging as the most influential. Higher values of this feature were predominantly associated with positive SHAP values, indicating an increased predicted probability of TZ-PCa. This GLSZM metric quantifies the prevalence of small, low grey-level zones after wavelet decomposition, capturing fine-scale fragmentation of T2 hypointense texture. TZ-PCa commonly shows infiltrative growth with increased cellularity and architectural distortion, which may manifest as more fragmented low-signal texture, whereas BPH typically forms nodules with mixed glandular components that more often appear as coarser, confluent regions even when T2-hypointense. GLSZM zone-size descriptors have been linked to PCa microstructural complexity and cellularity (29). This is consistent with known histopathological differences. Namely, TZ-PCa may appear as a relatively homogeneous, lenticular area of moderate T2 hypointensity due to compact cancerous tissue, whereas BPH reflects variable stromal and glandular components.

Although our combined model achieved performance comparable to prior studies based on lesion-specific ROIs, the whole-TZ approach offers a key advantage by eliminating the need for explicit tumor localization. Lesion-specific segmentation requires radiologists to delineate suspicious areas, a process prone to human error and particularly challenging for occult lesions with indistinct boundaries. Such limitations reduce the clinical applicability of lesion-based models. In contrast, whole-TZ segmentation reduces annotation workload and mitigates boundary-related uncertainties, thereby enhancing the feasibility of clinical application and simplifying the pipeline for potential AI deployment (30).

In practice, after routine bp-MRI acquisition, a radiologist delineates the whole transition zone on the axial slice corresponding to the largest lesion diameter. Radiomics features are automatically extracted and combined with PI-RADS score and tPSA to generate a predicted probability within minutes. This output can be used as a decision-support tool to assist biopsy recommendations rather than as a standalone diagnostic criterion. For threshold selection in deployment, we recommend an adjustable strategy tailored to clinical context and risk preferences. For example, lower thresholds (e.g., 0.3-0.5) in settings prioritizing avoidance of missed diagnoses, and higher thresholds (e.g., 0.6-0.7) to reduce unnecessary biopsies. This flexible approach aligns with current clinical practice and will be refined in future prospective studies.

Nonetheless, this study has several limitations. Despite including data from two centers, the retrospective design may have introduced selection bias. To further validate the performance of the combined model, a prospective study with a larger patient cohort is required. Manual segmentation is both subjective and time-consuming. Future work should explore semi-automatic or deep learning segmentation to address this limitation more effectively. Additionally, subgroup analyses of stromal and glandular BPH were not conducted. Expanding the dataset to enable evaluation of these TZ-BPH subtypes will be essential for refining model performance and enhancing clinical applicability. As the internal validation set was used for model selection, its performance may be subject to mild optimistic bias. We therefore place primary emphasis on the external validation results from an independent center, where the clinical-radiomics model achieved an AUC of 0.829.

## 5 Conclusion

We developed and externally validated an interpretable machine learning model that integrates bp-MRI derived radiomics features with clinical parameters. The combined model demonstrated effective diagnostic performance for TZ-PCa while providing insights into the model’s decision-making process. By enhancing transparency and interpretability, this approach may facilitate clinical adoption and support more personalized treatment options for patients.

## Data Availability

The original contributions presented in the study are included in the article/**Supplementary Material**. Further inquiries can be directed to the corresponding author.
