# Supplementary material for: Developing an interpretable clinical-radiomics machine learning model using whole transition zone MRI analysis for improving diagnosis of transition zone prostate cancer
Source: Front Oncol. 2026 Feb 11;16:1716482. doi: 10.3389/fonc.2026.1716482 (PMC12932199; doi:10.3389/fonc.2026.1716482)
Supplement: Supplementary file 1 [file DataSheet1.docx]

Supplementary Material

# Supplementary Figures and Tables

**Table S1** These details of the imaging sequence parameters for two hospitals

| Parameters | Center A | | | Center B | | |
| --- | --- | --- | --- | --- | --- | --- |
| MR scanner | GE SIGNA Voyager 1.5T | | | GE Discovery MR750 3.0T | | |
| Parameters | T1WI | T2WI | DWI | T1WI | T2WI | DWI |
| Sequence | TSE | TSE | EPI | VIBE | FSE | EPI |
| TR/TE (ms) | 541/15 | 4500/110 | 6900/100 | 843/12.2 | 11399.8/99.9 | 3417/53.7 |
| Slice thickness (mm) | 4 | 4 | 4 | 5 | 3 | 3 |
| Gap (mm) | 4 | 4 | 4 | 0 | 0 | 0 |


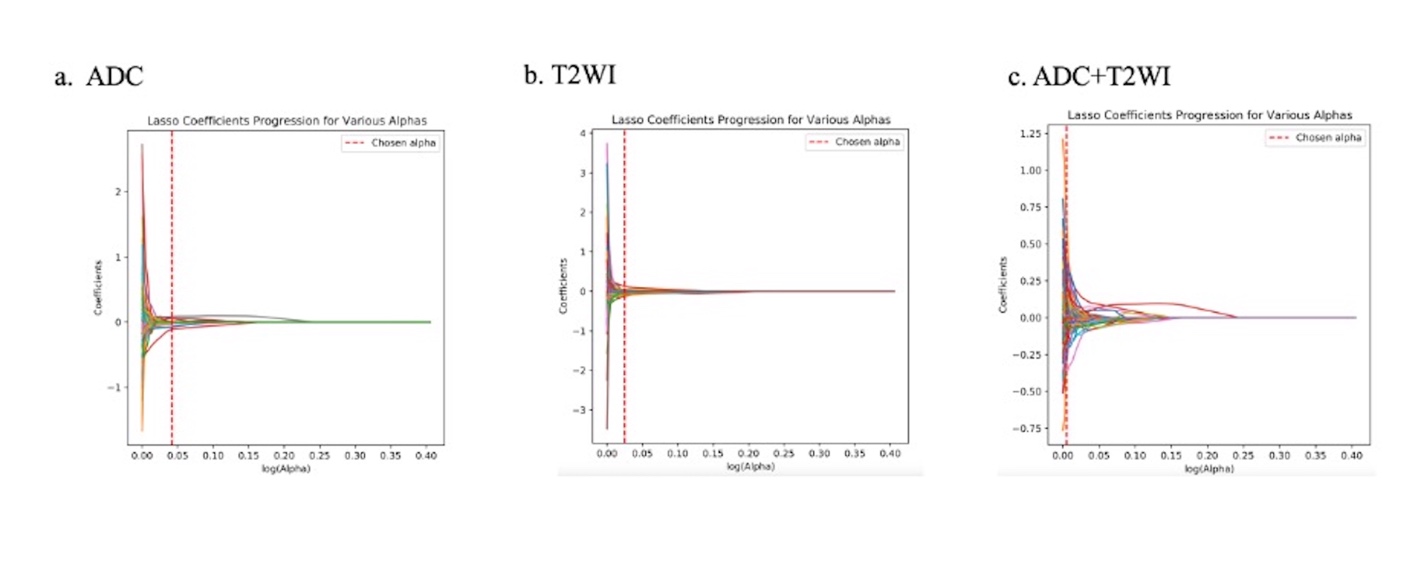


**Figure S1 Plot of Feature Screening Coefficients in Lasso Regression Analysis.** Tuning parameter (λ) selection in the LASSO model used ten-fold cross-validation via minimum criteria. The dotted vertical line represents theoptimal value. λ value of 0.0427 (a), 0.0251 (b) and 0.0050 (c) was chosen using ten-fold cross-validation.


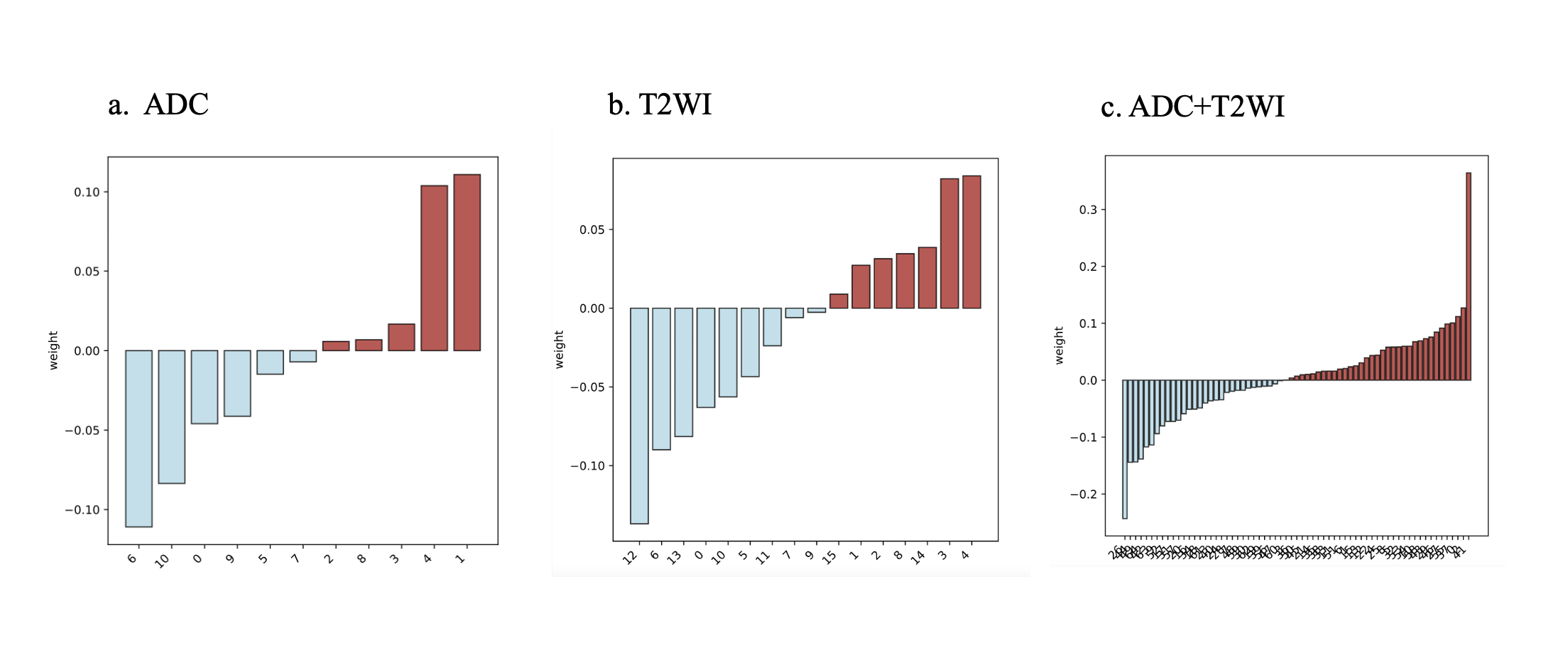


**Figure S2** **Histogram of feature weight proportion of LASSO regression.**

**
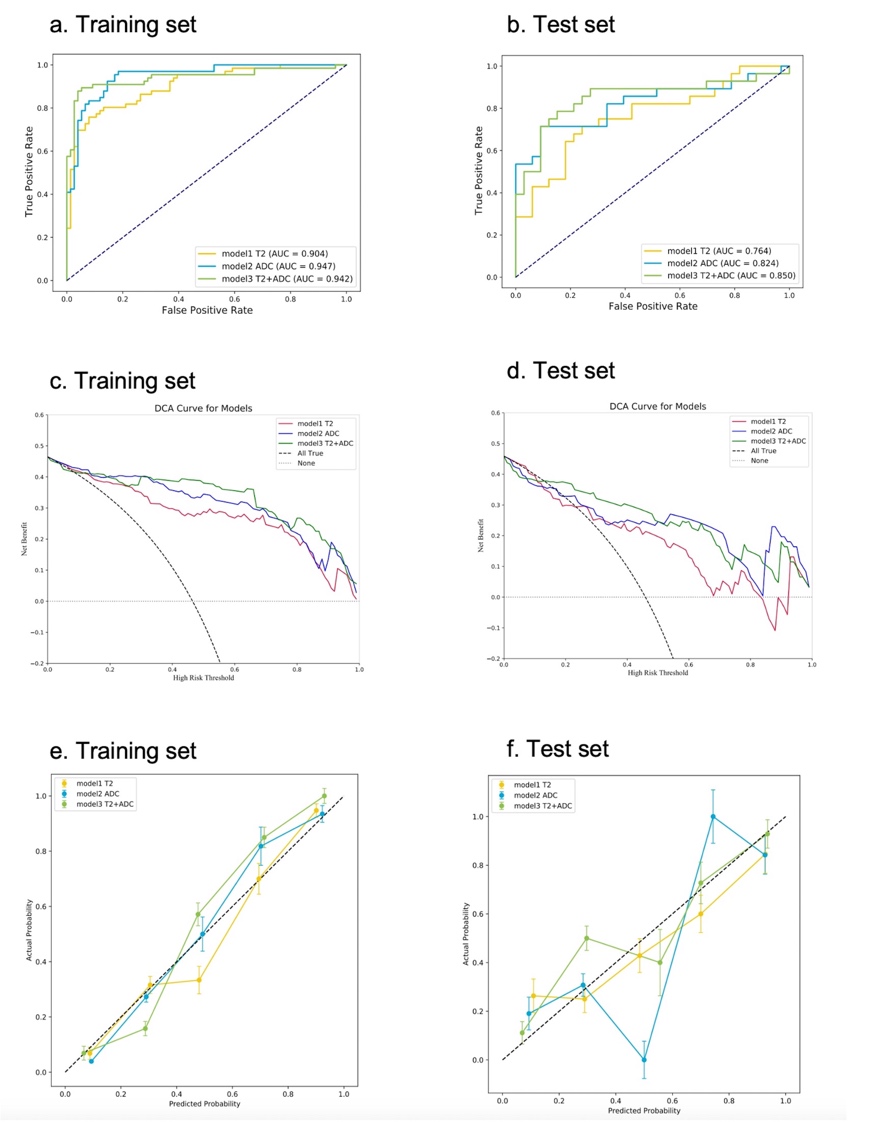
**

**Figure S3 ROC curves, decision curve analysis, calibration curves for the SVM Radiomics model in the training and internal validating sets.** The Receiver Operating Characteristic (ROC) curves of the SVM Radiomics model for both training and test sets can be seen in panels (a) and (b). (c) and (d) illustrate the decision curves of the SVM Radiomics model in the training and test groups. (e) and (f) illustrate the calibration curves of the SVM Radiomics model in the training and test groups.

# Supplementary Methods

## Image preprocessing

All MRI images data were exported from the PACS system in the original DICOM format, and then to mitigate bias resulting from differences in imaging equipment parameters, individual variations, and scanning conditions, MRI images underwent preprocessing. N4 bias field correction (N4ITK Bias Field Correction) and normalization methods were applied. N4 bias field correction, based on a nonparametric nonuniform intensity normalization algorithm, corrects low-frequency intensity inhomogeneity (bias field effect) in MRI images to improve image quality. Normalization adjusted the gray values of the images to a unified range (e.g., 0 to 1 or with a mean of 0 and a standard deviation of 1) to reduce intensity variations caused by differences in equipment and scanning conditions.

## Data dimension reduction methodology

Extracted texture features were standardized(z-score). z-score normalization to make the image intensities have the properties of a standard normal distribution with $\mu\text{=}1$ and $\sigma\text{=}0$, where $\mu$ was the mean value of the images, and was the standard deviation. The normalized values (also called z scores) of the image intensities (*x*) were $\sigma$calculated as follows:

$$z=\frac{x-\mu}{\sigma}$$
